# Association between morphometric measurements and disease progression in Cavalier King Charles Spaniels with preclinical degenerative mitral valve disease: A retrospective longitudinal study

**DOI:** 10.1371/journal.pone.0335420

**Published:** 2026-03-19

**Authors:** Sara Ghilardi, Fabio Maria Colombo, Mara Bagardi, Michele Polli, Maria Cristina Silvia Cozzi, Paola Giuseppina Brambilla

**Affiliations:** Department of Veterinary Medicine and Animal Science, University of Milan, Lodi, Italy; Universidade Federal de Minas Gerais, BRAZIL

## Abstract

Cavalier King Charles Spaniels (CKCS) are predisposed to a hereditable form of myxomatous mitral valve disease (MMVD) with a high incidence and a high risk of disease progression. Gender is recognized as a risk factor for the development of the disease in the breed. However, patient’s morphological traits associated with disease progression have not been identified. The aim of this study was to analyze the association between preclinical MMVD progression and morphometric features in CKCSs. This was a retrospective, time-to-event, longitudinal study. Medical records of 302 CKCSs were reviewed from April 2019 to January 2023. The final sample size counted 52 adult (≥ 1 year) MMVD-affected CKCSs classified as American College of Veterinary Internal Medicine (ACVIM) stage B1, with a minimum 24-month follow-up period and ≥ 2 follow-up examinations. At baseline examination, each dog underwent echocardiography and morphometric evaluation of the body, the head and the thorax; dogs were re-examined at 6- or 12-month intervals, and progression to ACVIM stage B2 was registered as the primary outcome. The median follow-up time was 1270.50 days (interquartile range (IQR, 25^th^ and 75^th^): 994.75–1525.50 days). In the time-to-event analysis, 17 (32.7%) dogs reached the endpoint, with a median time of 1548 days. Results from the Cox multiple regression analysis with inverse probability weighting (IPW) analysis showed that the following morphometric variables had an association (*p* < 0.05) with MMVD progression: thoracic length (hazard ratio (HR): 1.97, *p* = 0.002), thoracic circumference (HR: 0.61, *p* = 0.03), and thoracic index (HR: 0.86, *p* = 0.049). Data obtained from this study suggest that an association between mitral valve disease progression and thoracic morphology exists in MMVD-affected CKCSs classified as ACVIM stage B1. Morphological features should be considered along with other known risk factors for the breed when assessing the risk of MMVD progression in CKCSs.

## Introduction

Myxomatous mitral valve disease (MMVD) is the most common acquired canine heart disease, accounting for 75% of cardiac diseases in dogs [1]. Chronic MMVD undergoes a prolonged pre-clinical phase, with chamber dilation secondary to chronic mitral regurgitation (MR) being evident only later in life [1,2]. Certain breeds, such as the Cavalier King Charles Spaniel (CKCS) and the Dachshund, are believed to exhibit an inherited variant of MMVD, as they present a high prevalence of an early-onset form of MMVD, with diagnoses often occurring before 6–10 years of age, respectively [3–7]. In particular, the CKCS emerges as a breed with significantly higher incidence of disease and higher risk of disease progression [7,8]; as a result, extensive research has been dedicated to this breed, highlighting the importance of a multiple approach for early disease detection [9]. The prevailing hypothesis suggests that CKCSs may be affected by both the classic degenerative form of MMVD, which progresses slowly and leads to clinical signs eventually later in life, and a hereditary early-onset variant characterized by earlier manifestation and potentially severe morbidity and mortality [6,7,10]. This early-onset variant is believed to be a polygenic threshold trait [6,10–13]. Canine MMVD exhibits parallels to mitral valve prolapse (MVP) in humans, whose development is associated with genetic mutations [14,15]; however, to date little is known about MMVD-associated mutations in dogs, since studies conducted on the CKCS and the Dachshund did not report any genetic variant in the genes responsible for MVP in humans [16–18]. More recent studies have indicated that specific risk alleles for MMVD in CKCSs may reside within the Nebulette (NEBL) gene on the canine chromosome (CFA) 2; still, a comprehensive understanding of the genetic risk factors related to MMVD in this breed is far from being achieved [18–21].

It is critical to recognize that genetic predisposition is not the sole contributing factor predisposing to the development and progression of MMVD. In human medicine, a range of patients’ characteristics and environmental risk factors —such as race, gender, smoking habits, dietary patterns, body mass index, and systemic hypertension— have been acknowledged as influential in the progression of MVP [22]. Furthermore, human patients exhibiting MVP are frequently associated with some thoracic skeletal abnormalities (TSA), including scoliosis, pectus excavatum, and Marfan syndrome, and even in absence of severe forms of TSA, MVP individuals may be found with a concave-shaped chest wall (narrow antero-posterior thoracic diameter), so the relationship between thoracic morphology and MVP in humans is well established [23,24].

In CKCSs, gender has been recognized as a significant risk factor; males usually develop MMVD at a younger age and therefore exhibit a higher prevalence than females when adult [25]. Additionally, there are distinct differences in mitral valve morphology between healthy CKCSs and healthy dogs of other breeds. CKCSs typically possess a flatter mitral valve with reduced tenting and a smaller posterior leaflet. These morphological characteristics may subject the mitral valve to abnormal stress, thereby contributing to the early onset of MMVD in this breed [26]. At present, other environmental risk factors or patients’ characteristics associated with the progression of MMVD remain inadequately identified, hampering the setting of breed-specific management strategies for this condition. A recent study revealed that specific head and thoracic measurements are related to the severity of MR and to mitral annular dimensions in CKCSs [27]. This finding supports the hypothesis that canine morphology may serve as a risk factor for the development and progression of MMVD within this breed [27]. Similar results have been reported for the Dachshund, where gender was linked to the progression of MVP, and disease severity was related to specific thoracic dimensions and coat color types [4].

The role of canine morphology as a potential risk factor for the progression of this disease in dogs remains uncertain, since previous studies have not longitudinally examined the relationship between MMVD progression and morphometric measurements [4,27]. Therefore, this study aims to explore the association between the progression of MMVD and specific morphological characteristics (i.e., measurements of the head, the body, and the thorax) in preclinical MMVD-affected CKCSs. Exploring the relationship between MMVD progression and morphological features in this breed could help in better characterizing the disease, clarifying which dogs could potentially be predisposed to its faster-progression form. Furthermore, the identification of a specific morphotype associated with a higher risk of MMVD progression could help the breeders in setting specific management plans for this condition in the breed.

We hypothesized that morphotype may contribute to disease progression in the CKCS.

## Materials and methods

### Study design, setting and eligibility criteria

Following the Animal Research: Reporting of *In Vivo* Experiments (ARRIVE) guidelines, we conducted a retrospective, time-to-event, longitudinal study.

Medical records of privately owned CKCSs examined from April 2019 to January 2023 at the Department of Veterinary Medicine and Animal Sciences of the University of Milan were reviewed. Informed written consent was signed by the owners at the time of the first clinical examination, according to the University Veterinary Teaching Hospital guidelines. According to the current regulations of the University of Milan (“Regolamento dell’Organismo Preposto al Benessere degli Animali – D.R. 1061, 18/02/2025”), ethical approval is not required for retrospective studies on animals when no experimental procedures are involved and data have been collected for clinical purposes. Since all the procedures we performed were part of routine care, no ethical approval was required. The data were accessed for research purposes in September 2024.

Only adult MMVD-affected CKCSs classified as American College of Veterinary Internal Medicine (ACVIM) stage B1 were included in the study. The diagnosis of MMVD was made through transthoracic echocardiography by experienced veterinarians (a professor of veterinary cardiology with more than 20 years of experience or an ECVIM-CA cardiology resident-in-training and a PhD student in cardiology, both under direct supervision of the professor). MyLab50 Gold and MyLab OmegaVet ultrasound machines (Esaote, Genova, Italy) equipped with multi-frequency phased array probes (3.5–5 and 7.5–10 MHz, and 1–5 and 2–9 MHz, respectively) were used for the study; probes were chosen according to the patient’s size.

Dogs were classified according to the current ACVIM guidelines [28]. Specifically, MMVD stage B1 was defined as follows: detection of an abnormality of the mitral valve apparatus associated with MR in asymptomatic patients along with left atrium-to-aortic root ratio (LA/Ao) < 1.6 [29] and left ventricular internal diameter in diastole normalized for body weight (LVIDdN) < 1.7 [28,30].

Only adult CKCSs with a minimum 24-month follow-up period and with at least two clinical and echocardiographic evaluations were included in the study. Dogs were considered adult when older than one year [31]. Furthermore, only dogs with morphometric assessment of the head, body and thorax during baseline examination were included in the study [27]. This was possible since our research group started performing a morphometric evaluation of CKCSs in 2019 for another study [27]; since then, we continued with the canine morphology assessment as part of the breed screening program for the CKCS provided by the Veterinary Teaching Hospital.

The date of study entry corresponded to the first cardiologic examination when MMVD was diagnosed [28]. Baseline examination included registration of identification number to check for the geographical origin of the patient, clinical history, clinical evaluation with non-invasive blood pressure measurement and body condition score (BCS) registration [32], echocardiography, electrocardiogram, and the acquisition of morphometric measurements of the body, the head and the thorax. Following the WSAVA guidelines, dogs were classified according to their BCS as follows: “too thin”, BCS 1–3; “ideal”, BCS 4–5; “overweight”, BCS 6–7; “obese”, BCS 8–9 [32,33].

Exclusion criteria were the presence of congenital or acquired cardiac diseases different from MMVD, respiratory diseases, systemic diseases (e.g., metabolic or oncologic diseases), rhythm disturbances, systemic hypertension, or pulmonary hypertension. Furthermore, dogs with incomplete data records at baseline or follow-up examinations (e.g., absence of morphometric evaluation, lack of a clinical record with a clear statement of the ACVIM stage at follow-up) were excluded from the study.

A previous study conducted by our group examined morphometric variables in CKCSs with preclinical MMVD and their correlation with echocardiographic parameters of disease severity at a single time point [27]. The populations of these two studies partially overlap. However, due to differing study designs, inclusion criteria, follow-up protocols, and research objectives, the two studies were designed to tackle distinct research questions and must be considered differently.

### Clinical measurements

#### Echocardiographic data.

For this study, the echocardiographic parameters extracted from clinical records during baseline examination were: left atrium-to-aortic root ratio (LA/Ao) [29,30]; left ventricular internal diameter in end-diastole normalized for body weight (LVIDdN) [30]; MR severity (which was semi-quantitatively assessed through the maximal ratio of the regurgitant jet area signal to LA area (ARJ/LAA) ratio [34]); E wave peak velocity.

#### Morphometric measurements.

At baseline examination, the coat color type was registered (i.e., Blenheim, ruby, tricolor, and black and tan) following the breed standard set by the Italian Kennel Club (Ente Nazionale della Cinofilia Italiana, ENCI). Afterward, a morphometric evaluation of the body, the thorax and the head was performed following indications from a previous study on the same breed [27]. Two authors (SG and MB) consistently acquired data at baseline. For this study, the following morphometric measurements were considered for the analysis along with the coat color type: withers height, body index [(body length × 100)/ thoracic circumference], thoracic length and circumference, thoracic index [(thoracic width × 100)/ thoracic height], head angle, cephalic index [(head width × 100)/ total head length], and cranio-facial ratio (CFR) [(nose length/ head length) × 100] [27,35,36]. Supporting File 1 (S1 File) can be accessed for a detailed description of how we obtained each morphometric measurement.

### Follow-up examinations

Follow-up examinations were conducted at intervals of approximately 6 or 12 month, based on the recommendations of the primary clinician. The study endpoint was defined as the progression of the disease to ACVIM stage B2 [28]. According to the ACVIM guidelines, stage B2 was defined as follows: detection of an abnormality of the mitral valve apparatus associated with MR in asymptomatic patients along with a heart murmur ≥ 3/6, LA/Ao ≥ 1.6 [29], LVIDdN ≥ 1.7 [30], and a breed-adjusted radiographic vertebral heart score (VHS) > 10.5 [37,38].

If a dog did not progress to the ACVIM stage B2, it was censored at the date of its last follow-up examination. The analysis period concluded when a dog reached the endpoint or the censoring date.

### Statistical analysis

In this time-to-event study, the endpoint was considered progressing to MMVD ACVIM stage B2; therefore, “survival” must be interpreted as “not progressing to MMVD ACVIM stage B2”. All statistical analyses were performed in the “R” environment (R Core Team, 2024). The main analysis was to investigate the effect of the morphometric variables (coat color type, withers height, body index, thoracic length and circumference, thoracic index, head angle, cephalic index, CFR) on survival by proportional hazards Cox multiple regression with the R package “survival” [39]. The potential confounders (age, gender, weight, BCS, heart murmur intensity, E wave peak velocity, LA/Ao, LVIDdN, MR severity) were considered with the method of “Inverse Probability Weighting” (IPW) [40] and incorporated in the abovementioned model. As all the regressors (morphometric variables) of the model were considered as exposures, the IPW weights were estimated with the R package “mvGPS” which estimates multivariate IPW weights [41]. The effect size was presented as hazard ratios (HR). The assumption of proportionality of the hazard was tested.

Before building the abovementioned multiple regression model, the following preliminary analyses were conducted: an examination of the distribution of numeric variables, with normally-distributed variables being described as mean ± standard deviation, and non-normally distributed variables as median and interquartile range (IQR, 25^th^ and 75^th^); simple Cox regressions (one regressor a time considered), with and without IPW, to detect any relevant issue related to single regressors; analysis of correlation between regressors and collinearity testing to detect redundant regressors that would need to be removed from multiple Cox model.

Finally, Kaplan-Meier curves were built from the IPW Cox multiple regression model. Two illustrative curves predicted from Cox model were selected to visually show the effect on survival of the significant morphometric variables. The first predicted curve was calculated by setting the values of all regressors to their means, simulating a “mean” setting for the population. The second curve was calculated to simulate a “favorable” setting capable to improve survival in the population. This was done by setting the values of all non-relevant regressors to their means (non-statistically significant in the multiple Cox model), and by setting the values of all relevant regressors (statistically significant in the multiple Cox model) as follows: low (25^th^ percentile) values were set when their estimated HR was above 1 (unfavorable); high (75^th^ percentile) values were set when their estimated HR was below 1 (favorable). The aim was to build a setting in which selected morphometric variables with unfavorable effects were reversed, and selected morphometric variables with favorable effects were enhanced. Ultimately, this simulated a “favorable” setting capable of improving survival. Statistical significance was set at *p* < 0.05.

Supporting File 2 (S2 File) can be accessed for the full dataset.

## Results

### Baseline data

**Fig 1:**
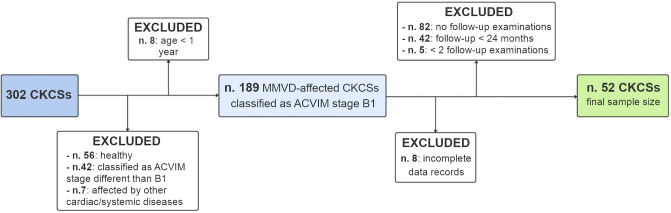
Flow chart showing the inclusion process of Cavalier King Charles Spaniels registered in our database between April 2019 and January 2023. Starting from 302 CKCSs, 8 were excluded because of their young age (< 1 year), 56 because they belonged to the ACVIM stage A (i.e., healthy dogs), while 42 CKCSs belonged to ACVIM stages different than B1. Seven dogs were affected by other cardiac or systemic diseases. Analyzing the follow-up data of the remaining 189 dogs, 82 were excluded because they had no follow-up evaluations, while 42 had a follow-up period < 24 months, and 5 dogs had a minimum of 24-month follow-up period but with only one follow-up examination. Eight dogs had incomplete data records. The final sample size counted 52 eligible CKCSs.

Clinical records of 302 CKCSs were evaluated during the study period. A flow diagram displaying the number of dogs excluded from the study after applying the exclusion criteria is presented in Fig 1 (Fig 1). Ultimately, the final study sample counted 52 adult MMVD-affected CKCSs classified as ACVIM stage B1, with a minimum of 24-month follow-up and ≥ 2 follow-up examinations. Of the 52 dogs involved in this study, 34 (65.4%) had also participated in a previous cross-sectional study on the same breed made by our research group [27]. The population counted 37 (71.2%) females and 15 (28.8%) males; the median age was 4.77 years (IQR: 3.43–6.24); the median weight was 9.35 kg (IQR: 8.15–10.33 kg), and 10 (19.2%) dogs were classified as “overweight” (specifically, 7 dogs had a BCS of 6/9 and 3 dogs had a BCS of 7/9) [32]. Thirty-nine of the included dogs (75.0%) had a body weight > 8 kg, which is the maximum weight limit accepted in the CKCS breed standard of the official Italian Kennel Club (ENCI). Considering the geographical origin of the dogs, 34 (65.4%) came from Italian breeders, 12 (23.1%) from French breeders, 2 (3.8%) from German breeders; it was not possible to establish the exact provenance of 4 (7.7%) subjects.

Heart murmur intensity was distributed as follows: 25 (48.1%) subjects did not present any heart murmur, 22 (42.3%) subjects had a systolic apical I/VI heart murmur, and 5 (9.6%) subjects had a systolic apical II/VI heart murmur. Table 1 reports echocardiographic parameters and morphometric variables distribution at baseline examination in 52 dogs enrolled for the final analyses.

**Table 1:** Selected baseline echocardiographic and morphologic data for the 52 adult Cavalier King Charles Spaniels (CKCSs) diagnosed with myxomatous mitral valve disease (MMVD) and classified as ACVIM stage B1 which were included in the final analyses. The results are expressed as frequency percentages (%) or median values with interquartile ranges (IQR), as the examined variables demonstrate a non-normal distribution.

Echocardiographic parameters								
LA/Ao			E wave peak velocity (m/s)		LVIDdN (cm/kg)		MR severity	
1.19 (1.10-1.31)			0.70 (0.64-0.77)		1.38 (1.31-1.48)		Mild n. 47 (90.4%) Moderate n. 5 (9.6%)	
**Morphometric measurements**								
**Coat color type**	**Withers height (cm)**	**Thoracic length (cm)**	**Thoracic circumference (cm)**	**Head angle (°)**	**Body index (%)**	**Thoracic index (%)**	**Cephalic index (%)**	**CFR (%)**
Blenheim n.32 (61.5%) Tricolor n.12 (23.1%) Other^*^ n.8 (15.4%)	29.80 (28.48-32.55)	20.75 (19.50-22.55)	47.25 (45.00-49.20)	120.00 (113.75-125.00)	71.95 (65.82-76.21)	81.07 (75.49-85.54)	69.90 (66.07-74.41)	41.71 (36.78-46.07)

CFR: craniofacial ratio; LA/Ao: left atrium-to-aortic root ratio; LVIDdN: left ventricular internal diameter in diastole normalized for body weight. In terms of mitral regurgitation (MR) severity, a regurgitant jet area signal to left atrium area (ARJ/LAA) ratio < 20–30% was classified as mild, while a ratio **≥** 20–30% and ≤ 70 was classified as moderate. Note that the coat colors ruby and black and tan were grouped due to the small sample size for each type.

The median body index in our population was 71.95% (IQR: 65.82–76.21%) and the median thoracic index was 81.07% (IQR: 75.49–85.54%), both indicating that the included CKCSs were mesomorphic [35]. On the other hand, cephalic index and CFR indicated a brachycephalic conformation of our population, with the median cephalic index being 69.90% (IQR: 66.07–74.41%) (brachycephalism: cephalic index > 50%) and the median CFR being 41.71% (IQR: 36.78–46.07%) [36].

### Outcome data

The median follow-up time in our population was 1270.50 days (IQR: 994.75–1525.50), and the median number of follow-up examinations was 3.00 (IQR: 2.00–4.00). In the time-to-event analysis, 17 (32.7%) dogs reached the endpoint (progression to MMVD ACVIM stage B2), with a median time of 1548 days. The others were censored at their last follow-up examination (n. 35, 67.3%). The first event was registered after 1126 days from baseline examination. Supporting Table 1 (S1 Table) reports clinical, echocardiographic and VHS outcome data distribution in the 17 dogs that reached the endpoint.

Before building the Cox multiple regression model, simple Cox regression analyses with and without IPW were conducted to detect any issue related to single regressors: none was detected. Furthermore, analysis of correlation between regressors was conducted, but no redundant regressor was identified.

**Fig 2:**
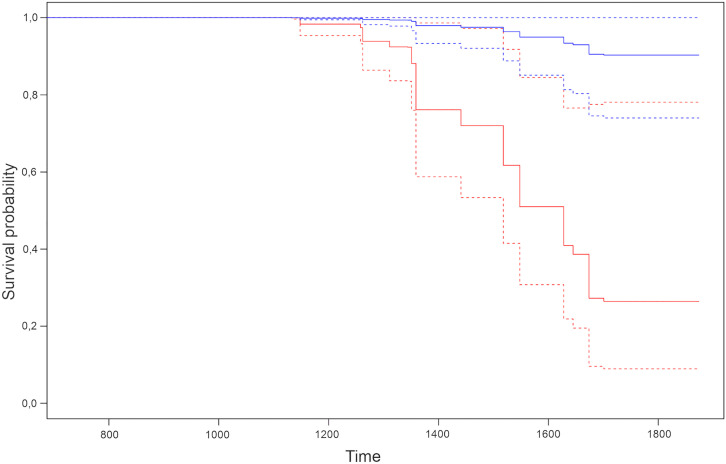
Kaplan-Meier curves built on two hypothetical subjects having different values of the three relevant morphometric variables.

In both the Cox simple and multiple regression analyses, coat color type did not show any association with MMVD progression in our selected population (*p* > 0.05), therefore it was excluded from further analyses. Results from the Cox multiple regression analysis with IPW showed that the following morphometric variables had an association (*p* < 0.05) with MMVD progression: thoracic length (HR: 1.97, *p* = 0.002), thoracic circumference (HR: 0.61, *p* = 0.03), and thoracic index (HR: 0.86, *p* = 0.049). Results are to be interpreted as follows: for each additional centimeter of thoracic length, the risk of disease progression increases by 97%; for each additional centimeter of thoracic circumference and for each one-unit increase in thoracic index, the risk of disease progression decreases by 39% and 14%, respectively. The assumption of proportionality of the hazards was satisfactorily tested. Since IPW was applied in the analysis, the confounders (age, gender, weight, BCS, heart murmur intensity, E wave peak velocity, LA/Ao, LVIDdN, MR severity) were incorporated in the statistical model. Fig 2 (Fig 2) shows two different predicted survival curves for our population, derived from the application of the IPW Cox multiple regression model.

Two Kaplan-Meier curves with their 95% confidence intervals (CI) are shown in the same plot. Solid lines represent Kaplan-Meier curves, while upper and lower CI limits are represented by dashed lines. Each curve was estimated from the predicted values of the Cox model used to analyze data.

Relevant morphometric variables were considered those independent variables that showed a significant effect in the Cox model (i.e., thoracic length, thoracic circumference and thoracic index).

The red Kaplan-Meier curve was estimated from a first hypothetical subject setting the relevant morphometric variables at their mean values, so this hypothetical subject represented a “mean” subject. The blue Kaplan-Meier curve was generated from a second hypothetical subject with relevant morphometric variables set at their “favorable” values [i.e., a low value (25^th^ percentile) for thoracic length, which has an adverse effect on survival with HR: 1.97, and high values (75^th^ percentile) for thoracic circumference and thoracic index, which have a protective effect on survival with HR: 0.61 and HR: 0.86, respectively]; so, this second hypothetical subject represents a subject with morphometric characteristics favorable for survival. The two curves are distinct, and the CI barely overlap, confirming the significant action of thoracic length, thoracic circumference, and thoracic index on survival: the “favored” hypothetical subject (blue) has a noticeable higher probability of not reaching the endpoint than the mean hypothetical subject (red).

## Discussion

This study represents the first investigation into the relationship between canine morphology and MMVD progression in adult CKCSs classified as ACVIM stage B1. The findings revealed a significant association between thoracic dimensions and disease progression in our sample group: thoracic length, thoracic circumference, and thoracic index were correlated with the advancement of MMVD to the ACVIM stage B2 in this breed. Specifically, dogs with a shorter thorax, greater thoracic circumference, and a higher thoracic index may exhibit a reduced risk of disease progression. Since higher values of the thoracic index, as well as a short thorax with a high circumference value, indicate a mesomorphic conformation [35], it could be speculated that a mesomorphic phenotype is associated with a lower probability of MMVD progression in the CKCS. Additionally, morphometric measurements of the head and the body, and coat color type did not significantly affect disease progression in our study sample.

Previous studies have examined the relationship between MMVD severity and canine morphology. In a previous research on CKCSs classified as ACVIM stage B1 [27], our group identified a link between brachycephalic conformation and a shorter anterior leaflet of the mitral valve with more severe MR. Additionally, our previous findings indicated a correlation between the diameters of the mitral valve annulus and both thoracic height and width and the relationship between thoracic circumference and the sphericity index [27]. Specifically, CKCSs with a wide and low-height thorax (characterized by a high thoracic index) exhibited larger mitral annular diameters during both systole and diastole, while dogs with greater thoracic circumferences demonstrated higher sphericity indexes [27].

When comparing those results to those obtained from the present study, some differences emerge: in our population, an association between head measurements and MMVD progression over time was not detected. Conversely, our results confirm that a relationship between thoracic measurements and MMVD, already suggested in that research, exists. Specifically, our previous study [27] indicated a correlation between a high thoracic index and larger mitral annular dimensions, which likely reflects a more dilated left ventricle, as dogs affected by MMVD typically exhibit a larger and rounder mitral valve annulus due to cardiac remodeling [42]. In contrast, these new analyses highlighted an association between a high thoracic index and a reduced risk of disease progression. The study by Olsen et al. identified a significant negative correlation between the severity of MVP and thoracic circumference, and a relationship between MVP and coat type in Dachshunds suffering from MMVD of varying severity [4]. While our population did not demonstrate an association between coat color type and the progression of MMVD, our findings regarding thoracic circumference align with those of the previous study: larger thoracic measurements correlate with milder forms of MVP in Dachshunds and a decreased risk of disease progression in our CKCSs cohort. Each of these studies highlights a link between thoracic dimensions and pathological features associated with MMVD in dogs. However, properly comparing the results of our longitudinal study and the previously mentioned cross-sectional studies is challenging.

Although 65.4% of the dogs included in this study were also part of a previously published cross-sectional study [27], the two investigations differ significantly in design, objectives, and analytical approach. The previous study was a cross-sectional analysis aimed at assessing the relationship between morphometric traits and echocardiographic indicators of MMVD severity at a single time point. In contrast, the current study utilized a longitudinal design with time-to-event analysis, allowing for an evaluation of how morphometric characteristics influence the progression of the disease in the breed over time. It is widely recognized that analyzing the same population using cross-sectional versus longitudinal designs can yield different, even contradictory, results. Cross-sectional studies provide a snapshot at a specific moment and are limited in establishing temporal or causal relationships. In contrast, longitudinal studies track changes within individuals over time, capturing dynamic processes, temporal ordering, and causal inference with greater precision [43–46]. Specifically, cross-sectional analyses may misrepresent mediation or causality and are more susceptible to temporal and confounding biases. Longitudinal data, on the other hand, offer better control over these biases and provide more accurate effect estimates, particularly when studying progression or outcomes [44,45]. These methodological differences likely account for the partially contrasting results between the two studies. Such differences underscore the importance of selecting the appropriate study design based on the research question; if the aim is to understand causal pathways or disease development over time, a longitudinal analysis is preferred [43–46]. Therefore, the differing outcomes between the two studies likely reflect differences in methodology and analytical approaches rather than inconsistencies in the data. We propose that the findings of the current study offer novel insights that complement—but do not contradict—the previous cross-sectional observations.

In human medicine, the relationship between chest conformation and MVP is well-established. The prevalence of MVP in individuals with TSA is significantly higher than in the general population, and chest wall conformation has been shown to influence cardiovascular outcomes in symptomatic patients with moderate MR secondary to MVP [23,47]. Notably, the chest wall shape, noninvasively assessed through the Modified Haller Index (MHI) (chest transverse diameter/ distance between sternum and spine), seems to be correlated to cardiovascular outcomes (i.e., hospitalization, mitral valve surgery, and cardiac or sudden death) in human patients with MVP and MR: an MHI ≥ 2.7 has 80% sensitivity and 100% specificity for predicting event-free survival in MVP-affected humans over a medium-term follow-up [44]. The MHI can be compared to the thoracic index that we measured in the present study [(thoracic width/ thoracic height) * 100], so our results agree with what is reported for humans [47].

The association between chest wall deformity, high MHI values, and MVP is not fully elucidated. The primary hypotheses include a) a defect in the embryogenesis of both the mitral valve and the bony thorax [48], and b) the continuous mechanical pressure from the deformity of the anterior chest wall, which may contribute to distortion and/or degeneration of the mitral valve [49]. Notably, human patients with an MHI greater than 2.5 and MVP often present with small cardiac chamber dimensions and exhibit normal left ventricular systolic and diastolic functions, along with optimal exercise tolerance. These factors may account for the favorable prognosis observed in these patients [50]. Consequently, a preliminary assessment of chest shape through the MHI method is recommended in human medicine to assist clinicians in evaluating the probability of MVP and identifying individuals at lower risk for cardiovascular events [47]. Similar considerations have never been made in veterinary medicine since we lack a systematic assessment of the thoracic conformation in our patients. However, our results suggest that the canine thoracic morphotype, assessed through the thoracic index, could help the clinician in identifying CKCSs that may be subjected to a lower risk of MMVD progression over time.

The study presents several limitations: a) the major limitation of this study is the relatively small sample size (n = 52), which may have greatly affected the statistical power of our analyses. Although the initial dataset included 302 dogs, the application of strict inclusion and exclusion criteria—designed to ensure diagnostic accuracy and homogeneity of the study population—resulted in a substantial reduction in the number of eligible subjects. Consequently, only 17 dogs reached the primary endpoint of progression from ACVIM stage B1 to B2, further limiting the number of events available for the time-to-event analysis. This low event rate increases the risk of type II errors and may diminish the robustness and generalizability of the findings; b) most of the dogs included in this study were bred in Italy. Considering the potential differences in breeding lines across countries, particularly regarding morphometric traits, our findings may be primarily applicable to the Italian population of Cavalier King Charles Spaniels; c) although we established a minimum follow-up period of 24 months, requiring at least two follow-up examinations as part of the inclusion criteria, and achieved a median follow-up duration of approximately 42 months, this timeframe may still be insufficient for a comprehensive evaluation of the progression of MMVD in CKCSs; d) the population’s median age at the time of inclusion was 4.77 years, suggesting that the enrolled dogs were relatively young. This may account for the fact that only 32.7% reached the MMVD ACVIM stage B2. Nonetheless, we included CKCSs of all adult ages, as they are known to be predisposed to an early-onset form of MMVD.

In conclusion, predicting MMVD progression in dogs is challenging, as the risk factors influencing the progression from mild to moderate/severe MR associated with cardiac remodeling remain uncertain. The data from this study suggest a connection between the progression of mitral valve disease and thoracic morphology in asymptomatic Italian CKCSs classified as ACVIM stage B1. These findings could provide practical insights for veterinary cardiologists who manage preclinical MMVD in CKCSs: evaluating thoracic conformation can serve as an additional, non-invasive tool to complement echocardiographic findings and established risk factors, and can be utilized to stratify the disease risk in this breed. Identifying dogs that may present an increased risk of disease progression could enhance clinical decision-making by providing personalized follow-up schedules and prioritizing more frequent monitoring for patients with unfavorable thoracic profiles. However, future studies with larger, multicenter cohorts of diverse geographical origins are necessary to validate these preliminary findings and to more definitively assess the prognostic value of morphometric parameters in the progression of mitral valve disease in Cavalier King Charles Spaniels. Such results could help establishing tailored management strategies for this condition within the breed.

## Supporting information

S1 Table Selected clinical, echocardiographic and radiographic outcome data for the 17 adult Cavalier King Charles Spaniels (CKCSs) diagnosed with myxomatous mitral valve disease (MMVD) and classified as American College of Veterinary Internal Medicine (ACVIM) stage B1 that reached the endpoint (disease progression to ACVIM stage B2). Categorical variables are expressed as frequencies (n) and percentages (%), numerical variables are expressed as mean ± standard deviation or median values with interquartile ranges (IQR), depending on their distribution (normal or non-normal, respectively).
(DOCX)

S1 File Detailed Materials & Methods’ section on how morphometric measurements were obtained. (DOCX)

S2 File Repository dataset. Excel file containing the full dataset used for statistical analyses. The file includes signalment data, clinical variables, echocardiographic and morphometric measurements, and follow-up data for all the enrolled Cavalier King Charles Spaniels (CKCSs). Variables are reported as described in the Methods section.
(XLSX)
