# Supplementary material for: Association between morphometric measurements and disease progression in Cavalier King Charles Spaniels with preclinical degenerative mitral valve disease: A retrospective longitudinal study
Source: PLoS One. 2026 Mar 19;21(3):e0335420. doi: 10.1371/journal.pone.0335420 (PMC13001908; doi:10.1371/journal.pone.0335420)
Supplement: S1 Table — Categorical variables are expressed as frequencies (n) and percentages (%), numerical variables are expressed as mean ± standard deviation or median values with interquartile ranges (IQR), depending on their distribution (normal or non-normal, respectively). (DOCX) [file pone.0335420.s001.docx]

**S1 Table.**

| Clinical variables | Gender distribution | Males n. 4 (23.5%)  Females n. 13 (76.5%) |
| --- | --- | --- |
|  | Age (years) | 9.55 ± 1.96 |
|  | Heart murmur intensity | III/VI n. 7 (41.2%)  IV/VI n. 7 (41.2%)  V-VI/VI n. 3 (17.6%) |
| Echocardiographic variables | LA/Ao | 1.79 ± 0.11 |
|  | E wave peak velocity (m/s) | 1.03 ± 0.13 |
|  | LVIDdN (cm/kg) | 2.00 (1.86-2.03) |
|  | MR severity | Moderate n. 12 (70.6%)  Severe n. 5 (29.4%) |
| Radiographic variables | VHS | 11.69 ± 0.39 |

LA/Ao: left atrium-to-aortic root ratio; LVIDdN: left ventricular internal diameter normalized for body weight; MR: mitral regurgitation; VHS: vertebral heart score
