# Supplementary material for: Association between morphometric measurements and disease progression in Cavalier King Charles Spaniels with preclinical degenerative mitral valve disease: A retrospective longitudinal study
Source: PLoS One. 2026 Mar 19;21(3):e0335420. doi: 10.1371/journal.pone.0335420 (PMC13001908; doi:10.1371/journal.pone.0335420)
Supplement: S1 File — (DOCX) [file pone.0335420.s002.docx]

**S1 File. Detailed Materials & Methods’ section on how morphometric measurements were obtained.**

For the morphometric evaluation, dogs were stacked by their owners on a table in the visit room. The operators that measured the dogs (SG and MB) always faced the dog on its left side to reduce systematic errors. The following tools were used: a small sliding gauge to measure head length, nose length and head width; a big custom-made sliding gauge to measure withers height, body length, and thoracic length, height and width; a measuring tape to measure thoracic circumference; a goniometer to obtain the head angle [1,2].

Morphometric measurements were obtained as follows:

- Withers height: from the withers (measured at the top of the shoulder blades) to the ground.
- Body length: from the most cranial point of the shoulder to the ischial tuberosity.
- Thoracic circumference: measured at the level of the first mammary nipples.
- Thoracic length: from the most cranial point of the shoulder to the midline of the last rib.
- Thoracic width: it is the biggest thoracic horizontal diameter measured just behind the shoulders.
- Thoracic height: it is the biggest thoracic vertical diameter and is obtained from the back to the sternum just behind the shoulders.
- Head angle: angle obtained by the intersection of a line tangent to the frontal region of the head (between the two orbits) and the line of the upper part of the nasal barrel.
- Head width: distance between the two zygomatic arches.
- Nose length: measured from the horizontal line joining the two inner corners of the eyelids to the cranial extremity of the truffle.
- Head length: measured from the occipital crest to the horizontal line joining the two inner corners of the eyelids.
- Total head length: obtained by summing the head length and nose length values [1,2].

1. Canton M. Dogs and Dog Breeds. 2nd ed. Porto Viro, Italy: Crepaldi A; 2011.
2. Bagardi M, Ghilardi S, Locatelli C, Bionda A, Polli M, Bussadori CM, et al. Influence of Morphometry on Echocardiographic Measurements in Cavalier King Charles Spaniels: An Inverse Probability Weighting Analysis. Vet Sci. 2021;8: 205.
